# 1-{Phen­yl[1-(*p*-tol­yl)ethyl­amino]meth­yl}-2-naphthol

**DOI:** 10.1107/S1600536808030365

**Published:** 2008-09-24

**Authors:** Yong Hua Li, Min Min Zhao, Yuan Zhang

**Affiliations:** a Ordered Matter Science Research Center, College of Chemistry and Chemical Engineering, Southeast University, Nanjing 211189, People’s Republic of China

## Abstract

The title compound, C_26_H_25_NO, was obtained *via* a one-pot synthesis from the reaction of 2-naphthol, 1-(*p*-tol­yl)ethyl­amine, *p*-toluene­sulfonic acid and benzaldehyde. There are three mol­ecules per asymmetric unit, all having similar conformations. There are intra­molecular O—H⋯N and C—H⋯O hydrogen bonds, with only van der Waals forces found between mol­ecules.

## Related literature

For background, see: Devi & Bhuyan (2004 ▶); Domling & Ugi (2000 ▶); Hulme & Gore (2003 ▶); Ugi (1962 ▶). For related literature, see: Liu *et al.* (2001 ▶).
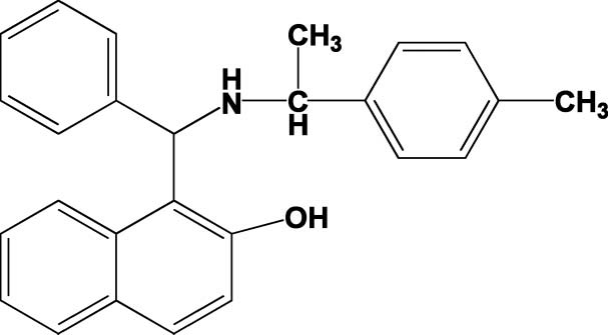


## Experimental

### 

#### Crystal data

C_26_H_25_NO
*M* *_r_* = 367.47
Triclinic, 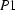

*a* = 9.3046 (19) Å
*b* = 13.126 (3) Å
*c* = 13.572 (3) Å
α = 88.14 (3)°
β = 89.99 (2)°
γ = 72.39 (3)°
*V* = 1579.0 (5) Å^3^
*Z* = 3
Mo *K*α radiation
μ = 0.07 mm^−1^
*T* = 292 (2) K
0.50 × 0.40 × 0.30 mm

#### Data collection

Rigaku SCXmini diffractometer
Absorption correction: multi-scan (*CrystalClear*; Rigaku, 2005 ▶) *T*_min_ = 0.950, *T*_max_ = 0.980
16610 measured reflections
7208 independent reflections
4449 reflections with *I* > 2σ(*I*)
*R*_int_ = 0.048

#### Refinement

*R*[*F* ^2^ > 2σ(*F* ^2^)] = 0.055
*wR*(*F* ^2^) = 0.136
*S* = 1.05
7208 reflections
760 parameters
3 restraints
H-atom parameters constrained
Δρ_max_ = 0.15 e Å^−3^
Δρ_min_ = −0.16 e Å^−3^

### 

Data collection: *CrystalClear* (Rigaku, 2005 ▶); cell refinement: *CrystalClear*; data reduction: *CrystalClear*; program(s) used to solve structure: *SHELXS97* (Sheldrick, 2008 ▶); program(s) used to refine structure: *SHELXL97* (Sheldrick, 2008 ▶); molecular graphics: *SHELXTL/PC* (Sheldrick, 2008 ▶); software used to prepare material for publication: *SHELXTL/PC*.

## Supplementary Material

Crystal structure: contains datablocks I, global. DOI: 10.1107/S1600536808030365/fl2220sup1.cif

Structure factors: contains datablocks I. DOI: 10.1107/S1600536808030365/fl2220Isup2.hkl

Additional supplementary materials:  crystallographic information; 3D view; checkCIF report

## Figures and Tables

**Table 1:** Hydrogen-bond geometry (Å, °)

*D*—H⋯*A*	*D*—H	H⋯*A*	*D*⋯*A*	*D*—H⋯*A*
O1—H1⋯N1	0.82	1.86	2.571 (4)	144
O2—H8⋯N2	0.82	1.92	2.629 (5)	144
O3—H2⋯N3	0.82	1.87	2.597 (5)	147
C6—H53*A*⋯O2	0.93	2.58	3.327 (6)	138

**Fig. 1.**
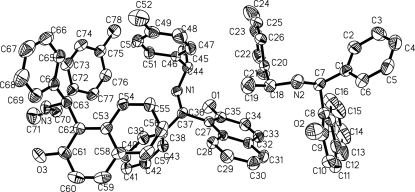
The molecular structure of the title compound, showing the atomic numbering scheme. Displacement ellipsoids are drawn at the 30% probability level.

## supplementary crystallographic information

### Comment

Multi-component reactions (MCRs) (Hulme & Gore, 2003; Ugi,1962) involving at least three starting materials in a one-pot reaction have attracted considerable attention in terms of saving both energy and raw materials (Devi & Bhuyan, 2004). Compared to conventional multi-step organic syntheses, MCRs have merits over multi-step reactions that include the simplicity of a one-pot procedure and the buildup of complex molecules (Domling & Ugi, 2000). Here we report the synthesis and crystal structure of the title compound (I, Fig. 1), obtained by a four-component condensation reaction as described in the experimental section.

I is an optically active derivative that crystallized with three molecules per asymmetric unit which is very rare. A few examples of similar compounds which contain two molecules per asymmetric unit have been reported (Liu *et al.*, 2001).

All three molecules in the asymmetric unit have the same relative conformation for both chiral carbon atoms. The dihedral angles between the rings A (C1–C6), B (C8–C17) and C (C20–C25) are A/B =71.90°, B/C =20.28°. The dihedral angles between the rings D (C38–C43), E (C27–C36) and F (C46–C51) are D/E =73.62°, E/F = 45.16° and the dihedral angles between the rings G (C64–C69), H (C53–C62) and I (C72–C77) are G/H =77.41°, H/I =62.04°. The three molecules are all stabilized by intramolecular O—H···N hydrogen bonding however, only one is involved in intramolecular C—H···O hydrogen bonds, no similar C—H···O distances are found in the other two molecules (Table 1). Intermolecular attractions are only on the order of Van der Waals forces.

### Experimental

Benzaldehyde (1.59 g, 0.015 mol) and *p*-toluenesulfonic acid (0.1 g) was added to 2-naphthol (2.16 g, 0.015 mol) without solvent. 1-(*p*-tolyl)ethylamine (2.025 g, 0.015 mol) was added dropwise with cooling to 0°C to the above mixture under nitrogen. The temperature was then gradually raised to 120°C over a period of hour and the mixture was stirred at this temperature for 24 h, then 20 ml of ethanol 95% was added, the precipitate was filtered and washed with a small amount of ethanol 95%. The title compound was isolated using column chromatography (Petroleum ether: ethyl acetate-5:1). Single crystals suitable for X-ray diffraction analysis were obtained from slow evaporation of an ethyl acetate solution.

### Refinement

H atoms were positioned geometrically and refined using a riding model, with C—H = 0.93–0.98 Å and *U*_iso_(H) = 1.2–1.5*U*_eq_(C). In the absence of significant anomalous scattering effects, 6529 Friedel pairs were merged.

### Crystal data

**Table:**

C_26_H_25_NO	*Z* = 3
*M**_r_* = 367.47	*F*(000) = 588
Triclinic, *P*1	*D*_x_ = 1.159 Mg m^−^^3^
Hall symbol: P 1	Mo *K*α radiation, λ = 0.71073 Å
*a* = 9.3046 (19) Å	Cell parameters from 7208 reflections
*b* = 13.126 (3) Å	θ = 3.0–27.5°
*c* = 13.572 (3) Å	µ = 0.07 mm^−^^1^
α = 88.14 (3)°	*T* = 292 K
β = 89.99 (2)°	Prism, colorless
γ = 72.39 (3)°	0.50 × 0.40 × 0.30 mm
*V* = 1579.0 (5) Å^3^	

### Data collection

**Table:**

Rigaku SCXmini diffractometer	7208 independent reflections
Radiation source: fine-focus sealed tube	4449 reflections with *I* > 2σ(*I*)
graphite	*R*_int_ = 0.048
Detector resolution: 13.6612 pixels mm^-1^	θ_max_ = 27.5°, θ_min_ = 3.0°
CCD p rofilefitting scans	*h* = −12→12
Absorption correction: multi-scan (CrystalClear; Rigaku, 2005)	*k* = −17→17
*T*_min_ = 0.950, *T*_max_ = 0.980	*l* = −17→17
16610 measured reflections	

### Refinement

**Table:**

Refinement on *F*^2^	Primary atom site location: structure-invariant direct methods
Least-squares matrix: full	Secondary atom site location: difference Fourier map
*R*[*F*^2^ > 2σ(*F*^2^)] = 0.055	Hydrogen site location: inferred from neighbouring sites
*wR*(*F*^2^) = 0.136	H-atom parameters constrained
*S* = 1.05	*w* = 1/[σ^2^(*F*_o_^2^) + (0.0531*P*)^2^ + 0.0528*P*] where *P* = (*F*_o_^2^ + 2*F*_c_^2^)/3
7208 reflections	(Δ/σ)_max_ < 0.001
760 parameters	Δρ_max_ = 0.15 e Å^−^^3^
3 restraints	Δρ_min_ = −0.16 e Å^−^^3^

### Special details

**Table:**

Geometry. All e.s.d.'s (except the e.s.d. in the dihedral angle between two l.s. planes) are estimated using the full covariance matrix. The cell e.s.d.'s are taken into account individually in the estimation of e.s.d.'s in distances, angles and torsion angles; correlations between e.s.d.'s in cell parameters are only used when they are defined by crystal symmetry. An approximate (isotropic) treatment of cell e.s.d.'s is used for estimating e.s.d.'s involving l.s. planes.
Refinement. Refinement of *F*^2^ against ALL reflections. The weighted *R*-factor *wR* and goodness of fit *S* are based on *F*^2^, conventional *R*-factors *R* are based on *F*, with *F* set to zero for negative *F*^2^. The threshold expression of *F*^2^ > σ(*F*^2^) is used only for calculating *R*-factors(gt) *etc*. and is not relevant to the choice of reflections for refinement. *R*-factors based on *F*^2^ are statistically about twice as large as those based on *F*, and *R*- factors based on ALL data will be even larger.

### Fractional atomic coordinates and isotropic or equivalent isotropic displacement parameters (Å^2^)

**Table:**

	*x*	*y*	*z*	*U*_iso_*/*U*_eq_	
C1	0.3155 (4)	0.7172 (3)	0.8864 (3)	0.0628 (9)	
C2	0.2500 (5)	0.6843 (4)	0.9671 (3)	0.0804 (12)	
H50A	0.1711	0.6557	0.9583	0.096*	
C3	0.3005 (7)	0.6934 (4)	1.0618 (4)	0.0998 (15)	
H75A	0.2539	0.6720	1.1158	0.120*	
C4	0.4167 (7)	0.7332 (4)	1.0759 (4)	0.0918 (15)	
H67A	0.4523	0.7367	1.1394	0.110*	
C5	0.4819 (6)	0.7682 (4)	0.9965 (4)	0.0867 (13)	
H61A	0.5593	0.7982	1.0059	0.104*	
C6	0.4315 (5)	0.7588 (3)	0.9024 (3)	0.0759 (11)	
H53A	0.4775	0.7812	0.8486	0.091*	
C7	0.2582 (4)	0.7047 (3)	0.7836 (3)	0.0640 (10)	
H30A	0.1699	0.6793	0.7913	0.077*	
C8	0.2098 (5)	0.8090 (3)	0.7229 (3)	0.0695 (10)	
C9	0.3141 (7)	0.8430 (4)	0.6695 (3)	0.0906 (14)	
C10	0.2735 (11)	0.9451 (6)	0.6209 (4)	0.116 (2)	
H73A	0.3460	0.9675	0.5871	0.140*	
C11	0.1332 (13)	1.0083 (6)	0.6237 (5)	0.131 (3)	
H76A	0.1100	1.0751	0.5917	0.157*	
C12	0.0190 (8)	0.9803 (4)	0.6716 (4)	0.1030 (18)	
C13	−0.1273 (12)	1.0453 (6)	0.6728 (6)	0.158 (4)	
H81A	−0.1519	1.1115	0.6395	0.190*	
C14	−0.2387 (11)	1.0159 (9)	0.7215 (6)	0.182 (5)	
H83A	−0.3369	1.0618	0.7215	0.218*	
C15	−0.2030 (8)	0.9166 (8)	0.7711 (5)	0.156 (3)	
H84A	−0.2781	0.8957	0.8035	0.187*	
C16	−0.0564 (6)	0.8488 (5)	0.7722 (4)	0.1069 (17)	
H72A	−0.0346	0.7832	0.8062	0.128*	
C17	0.0586 (6)	0.8762 (4)	0.7240 (3)	0.0815 (13)	
C18	0.3134 (4)	0.5779 (3)	0.6466 (3)	0.0664 (10)	
H99A	0.2650	0.6378	0.6009	0.080*	
C19	0.4459 (5)	0.5019 (4)	0.5929 (4)	0.0968 (15)	
H79A	0.5165	0.5391	0.5739	0.145*	
H79B	0.4093	0.4770	0.5351	0.145*	
H79C	0.4951	0.4420	0.6359	0.145*	
C20	0.1955 (4)	0.5256 (3)	0.6774 (2)	0.0573 (9)	
C21	0.0467 (5)	0.5712 (3)	0.6513 (3)	0.0695 (10)	
H54A	0.0181	0.6346	0.6135	0.083*	
C22	−0.0609 (5)	0.5239 (5)	0.6809 (3)	0.0864 (13)	
H51A	−0.1607	0.5561	0.6615	0.104*	
C23	−0.0267 (7)	0.4327 (5)	0.7369 (3)	0.0868 (14)	
C24	0.1216 (7)	0.3864 (4)	0.7642 (3)	0.0882 (14)	
H52A	0.1485	0.3237	0.8031	0.106*	
C25	0.2306 (5)	0.4318 (3)	0.7348 (3)	0.0700 (10)	
H27A	0.3303	0.3987	0.7539	0.084*	
C26	−0.1445 (9)	0.3811 (7)	0.7711 (5)	0.151 (3)	
H10D	−0.2419	0.4228	0.7463	0.227*	
H10E	−0.1461	0.3778	0.8418	0.227*	
H10F	−0.1202	0.3101	0.7468	0.227*	
C27	0.0607 (4)	0.6526 (3)	0.3775 (2)	0.0514 (8)	
C28	0.2086 (4)	0.6212 (3)	0.3414 (3)	0.0612 (9)	
H3A	0.2409	0.5591	0.3058	0.073*	
C29	0.3079 (5)	0.6790 (3)	0.3568 (3)	0.0753 (11)	
H47A	0.4052	0.6557	0.3320	0.090*	
C30	0.2615 (6)	0.7725 (4)	0.4098 (3)	0.0811 (12)	
H44A	0.3285	0.8110	0.4214	0.097*	
C31	0.1192 (5)	0.8072 (3)	0.4444 (3)	0.0746 (11)	
H20A	0.0893	0.8699	0.4792	0.090*	
C32	0.0139 (4)	0.7496 (3)	0.4285 (3)	0.0594 (9)	
C33	−0.1334 (5)	0.7844 (3)	0.4658 (3)	0.0693 (10)	
H18A	−0.1637	0.8467	0.5011	0.083*	
C34	−0.2299 (5)	0.7295 (3)	0.4514 (3)	0.0723 (11)	
H35A	−0.3274	0.7552	0.4754	0.087*	
C35	−0.1874 (4)	0.6327 (3)	0.4001 (3)	0.0603 (9)	
C36	−0.0418 (4)	0.5921 (3)	0.3643 (2)	0.0516 (8)	
C37	0.0086 (4)	0.4862 (3)	0.3122 (3)	0.0530 (8)	
H6A	0.1141	0.4508	0.3309	0.064*	
C38	0.0010 (4)	0.4984 (3)	0.2004 (3)	0.0514 (8)	
C39	0.1208 (4)	0.4455 (3)	0.1437 (3)	0.0696 (10)	
H36A	0.2089	0.4031	0.1745	0.084*	
C40	0.1140 (5)	0.4536 (4)	0.0429 (3)	0.0782 (11)	
H80A	0.1972	0.4178	0.0061	0.094*	
C41	−0.0152 (5)	0.5143 (3)	−0.0036 (3)	0.0721 (11)	
H38A	−0.0201	0.5200	−0.0720	0.086*	
C42	−0.1368 (5)	0.5663 (3)	0.0509 (3)	0.0740 (11)	
H46A	−0.2253	0.6070	0.0194	0.089*	
C43	−0.1292 (4)	0.5590 (3)	0.1527 (3)	0.0652 (10)	
H57A	−0.2125	0.5952	0.1893	0.078*	
C44	−0.0337 (4)	0.3634 (3)	0.4442 (3)	0.0628 (9)	
H32A	−0.0308	0.4192	0.4896	0.075*	
C45	−0.1560 (5)	0.3160 (4)	0.4792 (4)	0.0927 (14)	
H56A	−0.2519	0.3706	0.4768	0.139*	
H56B	−0.1343	0.2883	0.5457	0.139*	
H56C	−0.1591	0.2592	0.4373	0.139*	
C46	0.1198 (4)	0.2804 (3)	0.4471 (2)	0.0535 (8)	
C47	0.2259 (5)	0.2823 (3)	0.5164 (3)	0.0752 (11)	
H64A	0.2042	0.3380	0.5601	0.090*	
C48	0.3640 (5)	0.2044 (4)	0.5235 (4)	0.0903 (13)	
H71A	0.4328	0.2081	0.5719	0.108*	
C49	0.4011 (5)	0.1209 (4)	0.4594 (3)	0.0783 (12)	
C50	0.2946 (5)	0.1187 (3)	0.3913 (3)	0.0719 (11)	
H59A	0.3156	0.0621	0.3487	0.086*	
C51	0.1580 (4)	0.1963 (3)	0.3830 (3)	0.0660 (10)	
H22A	0.0901	0.1926	0.3340	0.079*	
C52	0.5525 (6)	0.0356 (5)	0.4674 (5)	0.129 (2)	
H82A	0.5590	−0.0161	0.4179	0.193*	
H82B	0.5632	0.0008	0.5315	0.193*	
H82C	0.6315	0.0680	0.4580	0.193*	
C53	0.5031 (3)	0.2743 (3)	0.1125 (3)	0.0528 (8)	
C54	0.4856 (4)	0.2479 (3)	0.2123 (3)	0.0571 (8)	
H4A	0.4625	0.1852	0.2281	0.069*	
C55	0.5015 (4)	0.3117 (3)	0.2868 (3)	0.0658 (10)	
H15A	0.4881	0.2923	0.3518	0.079*	
C56	0.5374 (4)	0.4049 (3)	0.2657 (4)	0.0761 (12)	
H41A	0.5478	0.4481	0.3165	0.091*	
C57	0.5572 (4)	0.4327 (3)	0.1722 (4)	0.0755 (12)	
H21A	0.5826	0.4950	0.1590	0.091*	
C58	0.5403 (4)	0.3698 (3)	0.0928 (3)	0.0637 (9)	
C59	0.5630 (5)	0.3966 (4)	−0.0060 (4)	0.0822 (13)	
H26A	0.5890	0.4585	−0.0203	0.099*	
C60	0.5482 (5)	0.3352 (4)	−0.0801 (4)	0.0822 (13)	
H43A	0.5657	0.3545	−0.1445	0.099*	
C61	0.5063 (4)	0.2417 (4)	−0.0614 (3)	0.0675 (10)	
C62	0.4835 (4)	0.2101 (3)	0.0338 (3)	0.0557 (8)	
C63	0.4476 (4)	0.1062 (3)	0.0570 (3)	0.0576 (9)	
H25A	0.3793	0.1178	0.1133	0.069*	
C64	0.5875 (4)	0.0136 (3)	0.0847 (3)	0.0622 (9)	
C65	0.5855 (6)	−0.0533 (3)	0.1655 (3)	0.0812 (12)	
H55A	0.4999	−0.0408	0.2042	0.097*	
C66	0.7132 (8)	−0.1398 (4)	0.1884 (4)	0.0960 (16)	
H70A	0.7131	−0.1833	0.2439	0.115*	
C67	0.8359 (7)	−0.1609 (4)	0.1313 (5)	0.1028 (17)	
H74A	0.9186	−0.2201	0.1462	0.123*	
C68	0.8397 (5)	−0.0961 (4)	0.0522 (5)	0.0941 (15)	
H68A	0.9257	−0.1106	0.0136	0.113*	
C69	0.7166 (5)	−0.0084 (3)	0.0284 (3)	0.0761 (11)	
H63A	0.7208	0.0358	−0.0258	0.091*	
C70	0.2047 (4)	0.1251 (3)	−0.0320 (3)	0.0685 (10)	
H37A	0.1860	0.2023	−0.0423	0.082*	
C71	0.1472 (6)	0.0848 (5)	−0.1227 (4)	0.1079 (17)	
H77A	0.2022	0.0973	−0.1794	0.162*	
H77B	0.0419	0.1221	−0.1323	0.162*	
H77C	0.1611	0.0096	−0.1141	0.162*	
C72	0.1207 (4)	0.1087 (3)	0.0598 (3)	0.0632 (9)	
C73	0.1590 (5)	0.0127 (3)	0.1132 (4)	0.0746 (11)	
H49A	0.2421	−0.0425	0.0939	0.090*	
C74	0.0789 (5)	−0.0041 (3)	0.1937 (4)	0.0751 (11)	
H48A	0.1100	−0.0693	0.2287	0.090*	
C75	−0.0481 (4)	0.0753 (3)	0.2232 (3)	0.0671 (10)	
C76	−0.0865 (4)	0.1706 (3)	0.1707 (3)	0.0699 (10)	
H19A	−0.1707	0.2252	0.1896	0.084*	
C77	−0.0053 (4)	0.1885 (3)	0.0910 (3)	0.0711 (11)	
H42A	−0.0348	0.2547	0.0576	0.085*	
C78	−0.1366 (5)	0.0563 (4)	0.3107 (4)	0.0927 (14)	
H10A	−0.0909	−0.0146	0.3379	0.139*	
H10B	−0.2383	0.0639	0.2905	0.139*	
H10C	−0.1371	0.1076	0.3598	0.139*	
N2	0.3758 (4)	0.6216 (3)	0.7309 (3)	0.0727 (8)	
H1A	0.4194	0.5675	0.7739	0.087*	
N3	0.3698 (4)	0.0750 (3)	−0.0274 (2)	0.0682 (8)	
H3C	0.3882	0.0036	−0.0233	0.082*	
N1	−0.0798 (3)	0.4155 (2)	0.3457 (2)	0.0610 (7)	
H4B	−0.0692	0.3642	0.3015	0.073*	
O1	−0.2945 (3)	0.5840 (2)	0.3874 (3)	0.0827 (8)	
H1	−0.2573	0.5266	0.3613	0.124*	
O3	0.4909 (4)	0.1879 (3)	−0.14207 (19)	0.0893 (9)	
H2	0.4449	0.1453	−0.1274	0.134*	
O2	0.4601 (4)	0.7828 (3)	0.6589 (3)	0.1158 (12)	
H8	0.4740	0.7246	0.6876	0.174*	

### Atomic displacement parameters (Å^2^)

**Table:**

	*U*^11^	*U*^22^	*U*^33^	*U*^12^	*U*^13^	*U*^23^
C1	0.069 (2)	0.050 (2)	0.068 (2)	−0.0162 (18)	−0.0044 (19)	0.0020 (18)
C2	0.088 (3)	0.083 (3)	0.074 (3)	−0.032 (2)	−0.005 (2)	0.002 (2)
C3	0.121 (4)	0.097 (4)	0.073 (3)	−0.022 (3)	−0.001 (3)	0.007 (3)
C4	0.107 (4)	0.069 (3)	0.085 (3)	−0.005 (3)	−0.031 (3)	−0.009 (3)
C5	0.087 (3)	0.063 (3)	0.109 (4)	−0.018 (2)	−0.020 (3)	−0.017 (3)
C6	0.079 (3)	0.065 (2)	0.087 (3)	−0.027 (2)	0.001 (2)	−0.009 (2)
C7	0.063 (2)	0.050 (2)	0.082 (3)	−0.0216 (18)	0.007 (2)	−0.0088 (18)
C8	0.089 (3)	0.069 (2)	0.058 (2)	−0.034 (2)	0.007 (2)	−0.0069 (19)
C9	0.127 (4)	0.085 (3)	0.077 (3)	−0.058 (3)	−0.002 (3)	−0.004 (2)
C10	0.201 (7)	0.095 (4)	0.080 (4)	−0.084 (5)	0.002 (4)	0.003 (3)
C11	0.237 (10)	0.088 (5)	0.079 (4)	−0.068 (6)	−0.021 (5)	−0.004 (3)
C12	0.155 (5)	0.075 (3)	0.062 (3)	−0.007 (4)	−0.026 (3)	−0.014 (3)
C13	0.219 (9)	0.090 (4)	0.098 (5)	0.057 (6)	−0.038 (5)	−0.027 (4)
C14	0.156 (7)	0.199 (10)	0.098 (5)	0.087 (7)	−0.025 (5)	−0.034 (6)
C15	0.104 (4)	0.209 (9)	0.100 (5)	0.037 (5)	−0.001 (4)	−0.018 (5)
C16	0.083 (3)	0.121 (4)	0.089 (3)	0.009 (3)	0.000 (3)	−0.008 (3)
C17	0.101 (3)	0.074 (3)	0.057 (2)	−0.007 (3)	−0.009 (2)	−0.013 (2)
C18	0.067 (2)	0.062 (2)	0.072 (2)	−0.0216 (19)	0.0000 (19)	−0.0044 (19)
C19	0.089 (3)	0.094 (3)	0.106 (4)	−0.024 (3)	0.034 (3)	−0.027 (3)
C20	0.070 (2)	0.056 (2)	0.0469 (19)	−0.0196 (18)	−0.0037 (17)	−0.0068 (16)
C21	0.071 (3)	0.071 (2)	0.062 (2)	−0.016 (2)	−0.0101 (19)	0.0071 (19)
C22	0.074 (3)	0.124 (4)	0.067 (3)	−0.038 (3)	−0.001 (2)	−0.009 (3)
C23	0.117 (4)	0.116 (4)	0.050 (2)	−0.069 (3)	0.006 (3)	−0.007 (3)
C24	0.137 (5)	0.083 (3)	0.056 (2)	−0.051 (3)	−0.002 (3)	0.009 (2)
C25	0.087 (3)	0.064 (2)	0.056 (2)	−0.019 (2)	−0.013 (2)	0.0050 (19)
C26	0.191 (7)	0.221 (8)	0.095 (4)	−0.144 (7)	0.015 (4)	−0.004 (4)
C27	0.058 (2)	0.0477 (18)	0.0436 (17)	−0.0085 (15)	−0.0042 (15)	−0.0026 (14)
C28	0.061 (2)	0.057 (2)	0.066 (2)	−0.0184 (18)	0.0022 (18)	−0.0063 (17)
C29	0.069 (2)	0.083 (3)	0.078 (3)	−0.029 (2)	−0.001 (2)	−0.004 (2)
C30	0.091 (3)	0.077 (3)	0.083 (3)	−0.037 (3)	−0.016 (3)	−0.004 (2)
C31	0.098 (3)	0.052 (2)	0.069 (2)	−0.015 (2)	−0.017 (2)	−0.0100 (19)
C32	0.070 (2)	0.051 (2)	0.0497 (19)	−0.0059 (18)	−0.0112 (17)	−0.0006 (16)
C33	0.078 (3)	0.056 (2)	0.058 (2)	0.005 (2)	−0.005 (2)	−0.0066 (18)
C34	0.062 (2)	0.068 (3)	0.071 (2)	0.006 (2)	0.0065 (19)	−0.005 (2)
C35	0.050 (2)	0.056 (2)	0.065 (2)	−0.0025 (17)	−0.0003 (17)	0.0030 (18)
C36	0.0481 (18)	0.0480 (19)	0.0523 (19)	−0.0051 (15)	−0.0015 (15)	−0.0005 (15)
C37	0.0464 (18)	0.0431 (18)	0.064 (2)	−0.0048 (14)	−0.0061 (15)	−0.0023 (15)
C38	0.0465 (17)	0.0447 (18)	0.060 (2)	−0.0084 (14)	−0.0026 (15)	−0.0055 (15)
C39	0.055 (2)	0.070 (3)	0.073 (3)	−0.0005 (18)	0.0018 (19)	−0.008 (2)
C40	0.076 (3)	0.081 (3)	0.068 (3)	−0.009 (2)	0.013 (2)	−0.015 (2)
C41	0.089 (3)	0.069 (3)	0.061 (2)	−0.027 (2)	0.001 (2)	−0.007 (2)
C42	0.075 (3)	0.074 (3)	0.066 (3)	−0.011 (2)	−0.010 (2)	−0.001 (2)
C43	0.054 (2)	0.067 (2)	0.067 (2)	−0.0070 (18)	−0.0020 (18)	−0.0086 (19)
C44	0.073 (2)	0.048 (2)	0.061 (2)	−0.0082 (18)	0.0039 (19)	−0.0026 (17)
C45	0.084 (3)	0.081 (3)	0.101 (3)	−0.008 (2)	0.024 (3)	0.018 (3)
C46	0.068 (2)	0.0451 (19)	0.0455 (18)	−0.0144 (16)	0.0030 (16)	−0.0029 (15)
C47	0.088 (3)	0.072 (3)	0.061 (2)	−0.016 (2)	−0.010 (2)	−0.013 (2)
C48	0.077 (3)	0.105 (4)	0.081 (3)	−0.016 (3)	−0.020 (2)	0.003 (3)
C49	0.066 (3)	0.081 (3)	0.076 (3)	−0.006 (2)	0.014 (2)	0.017 (2)
C50	0.080 (3)	0.059 (2)	0.067 (2)	−0.005 (2)	0.011 (2)	−0.0061 (19)
C51	0.074 (2)	0.062 (2)	0.058 (2)	−0.014 (2)	−0.0058 (19)	−0.0087 (18)
C52	0.072 (3)	0.136 (5)	0.144 (5)	0.015 (3)	0.022 (3)	0.034 (4)
C53	0.0386 (16)	0.0464 (18)	0.069 (2)	−0.0063 (14)	−0.0032 (15)	−0.0003 (16)
C54	0.053 (2)	0.053 (2)	0.063 (2)	−0.0121 (16)	0.0084 (16)	−0.0035 (17)
C55	0.065 (2)	0.059 (2)	0.071 (2)	−0.0135 (18)	0.0023 (19)	−0.0160 (19)
C56	0.063 (2)	0.059 (3)	0.103 (4)	−0.0125 (19)	−0.009 (2)	−0.019 (2)
C57	0.063 (2)	0.052 (2)	0.113 (4)	−0.0199 (19)	−0.014 (2)	0.001 (2)
C58	0.0474 (19)	0.058 (2)	0.085 (3)	−0.0155 (17)	−0.0019 (18)	0.0094 (19)
C59	0.069 (3)	0.076 (3)	0.106 (4)	−0.032 (2)	−0.007 (2)	0.028 (3)
C60	0.068 (3)	0.102 (3)	0.078 (3)	−0.030 (2)	0.001 (2)	0.028 (3)
C61	0.053 (2)	0.088 (3)	0.057 (2)	−0.0143 (19)	0.0048 (17)	0.005 (2)
C62	0.0451 (18)	0.058 (2)	0.060 (2)	−0.0095 (15)	0.0018 (15)	0.0013 (17)
C63	0.065 (2)	0.059 (2)	0.055 (2)	−0.0262 (18)	0.0045 (17)	−0.0107 (17)
C64	0.079 (3)	0.052 (2)	0.058 (2)	−0.0221 (19)	−0.0068 (19)	−0.0111 (17)
C65	0.118 (4)	0.061 (3)	0.065 (3)	−0.027 (3)	−0.009 (2)	−0.009 (2)
C66	0.150 (5)	0.057 (3)	0.079 (3)	−0.029 (3)	−0.038 (3)	0.004 (2)
C67	0.109 (4)	0.068 (3)	0.122 (5)	−0.013 (3)	−0.046 (4)	−0.005 (3)
C68	0.072 (3)	0.080 (3)	0.126 (4)	−0.014 (3)	−0.010 (3)	−0.019 (3)
C69	0.070 (3)	0.068 (3)	0.085 (3)	−0.014 (2)	−0.006 (2)	−0.003 (2)
C70	0.073 (3)	0.062 (2)	0.070 (2)	−0.020 (2)	−0.013 (2)	−0.0050 (19)
C71	0.108 (4)	0.127 (4)	0.090 (3)	−0.036 (3)	−0.030 (3)	−0.017 (3)
C72	0.060 (2)	0.053 (2)	0.077 (2)	−0.0167 (18)	−0.0134 (19)	−0.0044 (18)
C73	0.062 (2)	0.052 (2)	0.103 (3)	−0.0084 (18)	−0.001 (2)	−0.002 (2)
C74	0.068 (2)	0.055 (2)	0.100 (3)	−0.016 (2)	−0.006 (2)	0.005 (2)
C75	0.061 (2)	0.060 (2)	0.082 (3)	−0.0188 (19)	−0.012 (2)	−0.006 (2)
C76	0.051 (2)	0.069 (3)	0.087 (3)	−0.0127 (19)	−0.004 (2)	−0.009 (2)
C77	0.062 (2)	0.050 (2)	0.096 (3)	−0.0090 (18)	−0.018 (2)	−0.002 (2)
C78	0.081 (3)	0.091 (3)	0.108 (4)	−0.029 (3)	0.013 (3)	−0.007 (3)
N2	0.0649 (19)	0.071 (2)	0.083 (2)	−0.0212 (16)	−0.0017 (16)	−0.0115 (17)
N3	0.073 (2)	0.0654 (19)	0.0655 (19)	−0.0184 (16)	−0.0036 (15)	−0.0163 (15)
N1	0.0672 (18)	0.0509 (17)	0.0645 (18)	−0.0179 (14)	−0.0019 (15)	0.0030 (14)
O1	0.0465 (14)	0.0728 (18)	0.123 (3)	−0.0096 (13)	0.0032 (15)	−0.0069 (17)
O3	0.098 (2)	0.116 (3)	0.0550 (16)	−0.0339 (19)	0.0088 (15)	−0.0049 (17)
O2	0.105 (3)	0.140 (3)	0.128 (3)	−0.075 (3)	0.025 (2)	−0.007 (3)

### Geometric parameters (Å, °)

**Table:**

C1—C6	1.369 (5)	C42—C43	1.382 (5)
C1—C2	1.374 (6)	C42—H46A	0.9300
C1—C7	1.525 (5)	C43—H57A	0.9300
C2—C3	1.389 (7)	C44—N1	1.484 (5)
C2—H50A	0.9300	C44—C46	1.511 (5)
C3—C4	1.352 (7)	C44—C45	1.523 (6)
C3—H75A	0.9300	C44—H32A	0.9800
C4—C5	1.370 (7)	C45—H56A	0.9600
C4—H67A	0.9300	C45—H56B	0.9600
C5—C6	1.384 (6)	C45—H56C	0.9600
C5—H61A	0.9300	C46—C47	1.371 (5)
C6—H53A	0.9300	C46—C51	1.387 (5)
C7—N2	1.492 (5)	C47—C48	1.380 (6)
C7—C8	1.520 (6)	C47—H64A	0.9300
C7—H30A	0.9800	C48—C49	1.382 (7)
C8—C9	1.381 (6)	C48—H71A	0.9300
C8—C17	1.416 (6)	C49—C50	1.363 (6)
C9—O2	1.359 (6)	C49—C52	1.513 (6)
C9—C10	1.417 (8)	C50—C51	1.370 (5)
C10—C11	1.320 (9)	C50—H59A	0.9300
C10—H73A	0.9300	C51—H22A	0.9300
C11—C12	1.381 (9)	C52—H82A	0.9600
C11—H76A	0.9300	C52—H82B	0.9600
C12—C13	1.371 (10)	C52—H82C	0.9600
C12—C17	1.464 (7)	C53—C54	1.409 (5)
C13—C14	1.374 (13)	C53—C58	1.415 (5)
C13—H81A	0.9300	C53—C62	1.426 (5)
C14—C15	1.394 (13)	C54—C55	1.369 (5)
C14—H83A	0.9300	C54—H4A	0.9300
C15—C16	1.385 (8)	C55—C56	1.383 (6)
C15—H84A	0.9300	C55—H15A	0.9300
C16—C17	1.385 (7)	C56—C57	1.337 (6)
C16—H72A	0.9300	C56—H41A	0.9300
C18—N2	1.490 (5)	C57—C58	1.416 (6)
C18—C20	1.513 (5)	C57—H21A	0.9300
C18—C19	1.533 (6)	C58—C59	1.408 (6)
C18—H99A	0.9800	C59—C60	1.342 (7)
C19—H79A	0.9600	C59—H26A	0.9300
C19—H79B	0.9600	C60—C61	1.412 (6)
C19—H79C	0.9600	C60—H43A	0.9300
C20—C21	1.372 (5)	C61—O3	1.353 (5)
C20—C25	1.387 (5)	C61—C62	1.380 (5)
C21—C22	1.382 (6)	C62—C63	1.523 (5)
C21—H54A	0.9300	C63—N3	1.488 (4)
C22—C23	1.350 (7)	C63—C64	1.525 (5)
C22—H51A	0.9300	C63—H25A	0.9800
C23—C24	1.374 (7)	C64—C69	1.383 (6)
C23—C26	1.519 (7)	C64—C65	1.387 (6)
C24—C25	1.376 (6)	C65—C66	1.399 (7)
C24—H52A	0.9300	C65—H55A	0.9300
C25—H27A	0.9300	C66—C67	1.342 (8)
C26—H10D	0.9600	C66—H70A	0.9300
C26—H10E	0.9600	C67—C68	1.355 (8)
C26—H10F	0.9600	C67—H74A	0.9300
C27—C28	1.405 (5)	C68—C69	1.385 (6)
C27—C32	1.419 (5)	C68—H68A	0.9300
C27—C36	1.428 (5)	C69—H63A	0.9300
C28—C29	1.382 (5)	C70—N3	1.477 (5)
C28—H3A	0.9300	C70—C72	1.514 (6)
C29—C30	1.395 (6)	C70—C71	1.516 (6)
C29—H47A	0.9300	C70—H37A	0.9800
C30—C31	1.353 (6)	C71—H77A	0.9600
C30—H44A	0.9300	C71—H77B	0.9600
C31—C32	1.427 (6)	C71—H77C	0.9600
C31—H20A	0.9300	C72—C73	1.382 (5)
C32—C33	1.408 (6)	C72—C77	1.392 (5)
C33—C34	1.328 (6)	C73—C74	1.371 (6)
C33—H18A	0.9300	C73—H49A	0.9300
C34—C35	1.418 (6)	C74—C75	1.388 (5)
C34—H35A	0.9300	C74—H48A	0.9300
C35—O1	1.350 (5)	C75—C76	1.368 (6)
C35—C36	1.391 (5)	C75—C78	1.503 (6)
C36—C37	1.523 (5)	C76—C77	1.373 (6)
C37—N1	1.476 (4)	C76—H19A	0.9300
C37—C38	1.520 (5)	C77—H42A	0.9300
C37—H6A	0.9800	C78—H10A	0.9600
C38—C39	1.373 (5)	C78—H10B	0.9600
C38—C43	1.379 (5)	C78—H10C	0.9600
C39—C40	1.368 (6)	N2—H1A	0.9000
C39—H36A	0.9300	N3—H3C	0.9000
C40—C41	1.366 (6)	N1—H4B	0.9001
C40—H80A	0.9300	O1—H1	0.8200
C41—C42	1.363 (6)	O3—H2	0.8200
C41—H38A	0.9300	O2—H8	0.8200
			
C6—C1—C2	117.9 (4)	C38—C43—C42	120.5 (3)
C6—C1—C7	123.0 (4)	C38—C43—H57A	119.8
C2—C1—C7	119.1 (3)	C42—C43—H57A	119.8
C1—C2—C3	120.7 (4)	N1—C44—C46	114.4 (3)
C1—C2—H50A	119.7	N1—C44—C45	107.8 (3)
C3—C2—H50A	119.7	C46—C44—C45	111.4 (3)
C4—C3—C2	120.5 (5)	N1—C44—H32A	107.7
C4—C3—H75A	119.7	C46—C44—H32A	107.7
C2—C3—H75A	119.7	C45—C44—H32A	107.7
C3—C4—C5	119.8 (5)	C44—C45—H56A	109.5
C3—C4—H67A	120.1	C44—C45—H56B	109.5
C5—C4—H67A	120.1	H56A—C45—H56B	109.5
C4—C5—C6	119.5 (4)	C44—C45—H56C	109.5
C4—C5—H61A	120.3	H56A—C45—H56C	109.5
C6—C5—H61A	120.3	H56B—C45—H56C	109.5
C1—C6—C5	121.7 (4)	C47—C46—C51	116.8 (3)
C1—C6—H53A	119.2	C47—C46—C44	121.2 (3)
C5—C6—H53A	119.2	C51—C46—C44	122.0 (3)
N2—C7—C8	110.6 (3)	C46—C47—C48	122.1 (4)
N2—C7—C1	109.7 (3)	C46—C47—H64A	118.9
C8—C7—C1	113.1 (3)	C48—C47—H64A	118.9
N2—C7—H30A	107.8	C47—C48—C49	120.7 (4)
C8—C7—H30A	107.8	C47—C48—H71A	119.7
C1—C7—H30A	107.8	C49—C48—H71A	119.7
C9—C8—C17	118.6 (4)	C50—C49—C48	117.0 (4)
C9—C8—C7	120.5 (4)	C50—C49—C52	122.4 (5)
C17—C8—C7	120.8 (4)	C48—C49—C52	120.5 (5)
O2—C9—C8	123.6 (4)	C49—C50—C51	122.7 (4)
O2—C9—C10	115.4 (6)	C49—C50—H59A	118.7
C8—C9—C10	121.0 (6)	C51—C50—H59A	118.7
C11—C10—C9	120.2 (7)	C50—C51—C46	120.7 (4)
C11—C10—H73A	119.9	C50—C51—H22A	119.7
C9—C10—H73A	119.9	C46—C51—H22A	119.7
C10—C11—C12	123.3 (6)	C49—C52—H82A	109.5
C10—C11—H76A	118.3	C49—C52—H82B	109.5
C12—C11—H76A	118.3	H82A—C52—H82B	109.5
C13—C12—C11	123.2 (8)	C49—C52—H82C	109.5
C13—C12—C17	119.2 (7)	H82A—C52—H82C	109.5
C11—C12—C17	117.5 (6)	H82B—C52—H82C	109.5
C12—C13—C14	122.1 (8)	C54—C53—C58	116.5 (3)
C12—C13—H81A	118.9	C54—C53—C62	122.9 (3)
C14—C13—H81A	118.9	C58—C53—C62	120.6 (3)
C13—C14—C15	119.3 (7)	C55—C54—C53	122.1 (4)
C13—C14—H83A	120.3	C55—C54—H4A	119.0
C15—C14—H83A	120.3	C53—C54—H4A	119.0
C16—C15—C14	120.3 (8)	C54—C55—C56	120.3 (4)
C16—C15—H84A	119.8	C54—C55—H15A	119.8
C14—C15—H84A	119.8	C56—C55—H15A	119.8
C15—C16—C17	121.8 (7)	C57—C56—C55	119.9 (4)
C15—C16—H72A	119.1	C57—C56—H41A	120.0
C17—C16—H72A	119.1	C55—C56—H41A	120.0
C16—C17—C8	123.6 (4)	C56—C57—C58	121.7 (4)
C16—C17—C12	117.2 (5)	C56—C57—H21A	119.2
C8—C17—C12	119.2 (5)	C58—C57—H21A	119.2
N2—C18—C20	113.1 (3)	C59—C58—C53	117.9 (4)
N2—C18—C19	107.9 (3)	C59—C58—C57	122.6 (4)
C20—C18—C19	112.7 (3)	C53—C58—C57	119.5 (4)
N2—C18—H99A	107.6	C60—C59—C58	121.7 (4)
C20—C18—H99A	107.6	C60—C59—H26A	119.1
C19—C18—H99A	107.6	C58—C59—H26A	119.1
C18—C19—H79A	109.5	C59—C60—C61	120.7 (4)
C18—C19—H79B	109.5	C59—C60—H43A	119.7
H79A—C19—H79B	109.5	C61—C60—H43A	119.7
C18—C19—H79C	109.5	O3—C61—C62	124.0 (4)
H79A—C19—H79C	109.5	O3—C61—C60	115.4 (4)
H79B—C19—H79C	109.5	C62—C61—C60	120.6 (4)
C21—C20—C25	116.9 (4)	C61—C62—C53	118.4 (3)
C21—C20—C18	120.8 (3)	C61—C62—C63	121.9 (3)
C25—C20—C18	122.2 (3)	C53—C62—C63	119.6 (3)
C20—C21—C22	120.6 (4)	N3—C63—C62	111.4 (3)
C20—C21—H54A	119.7	N3—C63—C64	108.8 (3)
C22—C21—H54A	119.7	C62—C63—C64	112.7 (3)
C23—C22—C21	122.4 (5)	N3—C63—H25A	107.9
C23—C22—H51A	118.8	C62—C63—H25A	107.9
C21—C22—H51A	118.8	C64—C63—H25A	107.9
C22—C23—C24	117.7 (4)	C69—C64—C65	118.4 (4)
C22—C23—C26	122.8 (6)	C69—C64—C63	121.4 (3)
C24—C23—C26	119.5 (5)	C65—C64—C63	120.2 (4)
C23—C24—C25	120.7 (4)	C64—C65—C66	119.6 (5)
C23—C24—H52A	119.6	C64—C65—H55A	120.2
C25—C24—H52A	119.6	C66—C65—H55A	120.2
C24—C25—C20	121.6 (4)	C67—C66—C65	120.9 (5)
C24—C25—H27A	119.2	C67—C66—H70A	119.6
C20—C25—H27A	119.2	C65—C66—H70A	119.6
C23—C26—H10D	109.5	C66—C67—C68	120.2 (5)
C23—C26—H10E	109.5	C66—C67—H74A	119.9
H10D—C26—H10E	109.5	C68—C67—H74A	119.9
C23—C26—H10F	109.5	C67—C68—C69	120.6 (5)
H10D—C26—H10F	109.5	C67—C68—H68A	119.7
H10E—C26—H10F	109.5	C69—C68—H68A	119.7
C28—C27—C32	117.1 (3)	C64—C69—C68	120.3 (4)
C28—C27—C36	123.0 (3)	C64—C69—H63A	119.8
C32—C27—C36	119.9 (3)	C68—C69—H63A	119.8
C29—C28—C27	122.5 (4)	N3—C70—C72	115.3 (3)
C29—C28—H3A	118.8	N3—C70—C71	107.3 (4)
C27—C28—H3A	118.8	C72—C70—C71	111.9 (4)
C28—C29—C30	119.6 (4)	N3—C70—H37A	107.4
C28—C29—H47A	120.2	C72—C70—H37A	107.4
C30—C29—H47A	120.2	C71—C70—H37A	107.4
C31—C30—C29	120.1 (4)	C70—C71—H77A	109.5
C31—C30—H44A	119.9	C70—C71—H77B	109.5
C29—C30—H44A	119.9	H77A—C71—H77B	109.5
C30—C31—C32	121.3 (4)	C70—C71—H77C	109.5
C30—C31—H20A	119.3	H77A—C71—H77C	109.5
C32—C31—H20A	119.3	H77B—C71—H77C	109.5
C33—C32—C27	119.1 (4)	C73—C72—C77	116.7 (4)
C33—C32—C31	121.6 (4)	C73—C72—C70	122.2 (4)
C27—C32—C31	119.3 (3)	C77—C72—C70	121.0 (3)
C34—C33—C32	121.1 (4)	C74—C73—C72	122.4 (4)
C34—C33—H18A	119.5	C74—C73—H49A	118.8
C32—C33—H18A	119.5	C72—C73—H49A	118.8
C33—C34—C35	121.4 (4)	C73—C74—C75	120.5 (4)
C33—C34—H35A	119.3	C73—C74—H48A	119.8
C35—C34—H35A	119.3	C75—C74—H48A	119.8
O1—C35—C36	122.7 (3)	C76—C75—C74	117.4 (4)
O1—C35—C34	116.9 (3)	C76—C75—C78	122.2 (4)
C36—C35—C34	120.4 (4)	C74—C75—C78	120.4 (4)
C35—C36—C27	118.2 (3)	C75—C76—C77	122.4 (4)
C35—C36—C37	121.6 (3)	C75—C76—H19A	118.8
C27—C36—C37	120.3 (3)	C77—C76—H19A	118.8
N1—C37—C38	109.6 (3)	C76—C77—C72	120.7 (4)
N1—C37—C36	110.7 (3)	C76—C77—H42A	119.7
C38—C37—C36	113.8 (3)	C72—C77—H42A	119.7
N1—C37—H6A	107.5	C75—C78—H10A	109.5
C38—C37—H6A	107.5	C75—C78—H10B	109.5
C36—C37—H6A	107.5	H10A—C78—H10B	109.5
C39—C38—C43	118.0 (3)	C75—C78—H10C	109.5
C39—C38—C37	121.1 (3)	H10A—C78—H10C	109.5
C43—C38—C37	120.9 (3)	H10B—C78—H10C	109.5
C40—C39—C38	121.6 (4)	C18—N2—C7	112.9 (3)
C40—C39—H36A	119.2	C18—N2—H1A	108.6
C38—C39—H36A	119.2	C7—N2—H1A	108.5
C41—C40—C39	120.0 (4)	C70—N3—C63	115.2 (3)
C41—C40—H80A	120.0	C70—N3—H3C	107.9
C39—C40—H80A	120.0	C63—N3—H3C	108.0
C42—C41—C40	119.7 (4)	C37—N1—C44	114.0 (3)
C42—C41—H38A	120.2	C37—N1—H4B	108.4
C40—C41—H38A	120.2	C44—N1—H4B	108.4
C41—C42—C43	120.3 (4)	C35—O1—H1	109.5
C41—C42—H46A	119.8	C61—O3—H2	109.5
C43—C42—H46A	119.8	C9—O2—H8	109.5
			
C6—C1—C2—C3	0.1 (6)	C37—C38—C39—C40	178.3 (4)
C7—C1—C2—C3	179.3 (4)	C38—C39—C40—C41	−1.0 (7)
C1—C2—C3—C4	−1.1 (7)	C39—C40—C41—C42	−0.1 (6)
C2—C3—C4—C5	2.3 (7)	C40—C41—C42—C43	0.8 (6)
C3—C4—C5—C6	−2.4 (7)	C39—C38—C43—C42	−0.6 (5)
C2—C1—C6—C5	−0.2 (6)	C37—C38—C43—C42	−177.5 (3)
C7—C1—C6—C5	−179.4 (4)	C41—C42—C43—C38	−0.4 (6)
C4—C5—C6—C1	1.4 (6)	N1—C44—C46—C47	129.5 (4)
C6—C1—C7—N2	67.8 (4)	C45—C44—C46—C47	−107.9 (4)
C2—C1—C7—N2	−111.4 (4)	N1—C44—C46—C51	−53.4 (5)
C6—C1—C7—C8	−56.2 (5)	C45—C44—C46—C51	69.2 (5)
C2—C1—C7—C8	124.6 (4)	C51—C46—C47—C48	−0.6 (6)
N2—C7—C8—C9	−38.1 (5)	C44—C46—C47—C48	176.7 (4)
C1—C7—C8—C9	85.4 (5)	C46—C47—C48—C49	0.6 (7)
N2—C7—C8—C17	145.2 (4)	C47—C48—C49—C50	−1.2 (6)
C1—C7—C8—C17	−91.3 (4)	C47—C48—C49—C52	−180.0 (5)
C17—C8—C9—O2	−176.4 (4)	C48—C49—C50—C51	2.0 (6)
C7—C8—C9—O2	6.8 (6)	C52—C49—C50—C51	−179.3 (4)
C17—C8—C9—C10	3.3 (6)	C49—C50—C51—C46	−2.1 (6)
C7—C8—C9—C10	−173.4 (4)	C47—C46—C51—C50	1.3 (6)
O2—C9—C10—C11	177.7 (5)	C44—C46—C51—C50	−176.0 (3)
C8—C9—C10—C11	−2.1 (8)	C58—C53—C54—C55	0.9 (5)
C9—C10—C11—C12	−0.6 (10)	C62—C53—C54—C55	−178.5 (3)
C10—C11—C12—C13	−178.7 (6)	C53—C54—C55—C56	−0.7 (5)
C10—C11—C12—C17	1.9 (8)	C54—C55—C56—C57	−0.2 (6)
C11—C12—C13—C14	−179.5 (7)	C55—C56—C57—C58	0.9 (6)
C17—C12—C13—C14	−0.2 (10)	C54—C53—C58—C59	178.1 (3)
C12—C13—C14—C15	−0.4 (12)	C62—C53—C58—C59	−2.5 (5)
C13—C14—C15—C16	0.8 (12)	C54—C53—C58—C57	−0.2 (5)
C14—C15—C16—C17	−0.6 (10)	C62—C53—C58—C57	179.2 (3)
C15—C16—C17—C8	−179.7 (5)	C56—C57—C58—C59	−178.9 (4)
C15—C16—C17—C12	0.0 (8)	C56—C57—C58—C53	−0.7 (6)
C9—C8—C17—C16	177.7 (4)	C53—C58—C59—C60	0.9 (6)
C7—C8—C17—C16	−5.6 (6)	C57—C58—C59—C60	179.2 (4)
C9—C8—C17—C12	−2.0 (6)	C58—C59—C60—C61	1.1 (6)
C7—C8—C17—C12	174.7 (4)	C59—C60—C61—O3	178.3 (4)
C13—C12—C17—C16	0.4 (7)	C59—C60—C61—C62	−1.7 (6)
C11—C12—C17—C16	179.8 (5)	O3—C61—C62—C53	−179.8 (3)
C13—C12—C17—C8	−179.9 (5)	C60—C61—C62—C53	0.1 (5)
C11—C12—C17—C8	−0.5 (6)	O3—C61—C62—C63	3.8 (5)
N2—C18—C20—C21	110.8 (4)	C60—C61—C62—C63	−176.2 (3)
C19—C18—C20—C21	−126.5 (4)	C54—C53—C62—C61	−178.7 (3)
N2—C18—C20—C25	−67.5 (4)	C58—C53—C62—C61	2.0 (5)
C19—C18—C20—C25	55.2 (5)	C54—C53—C62—C63	−2.3 (5)
C25—C20—C21—C22	−0.6 (6)	C58—C53—C62—C63	178.4 (3)
C18—C20—C21—C22	−179.1 (4)	C61—C62—C63—N3	−27.7 (4)
C20—C21—C22—C23	0.8 (7)	C53—C62—C63—N3	156.1 (3)
C21—C22—C23—C24	−0.3 (7)	C61—C62—C63—C64	95.0 (4)
C21—C22—C23—C26	179.0 (5)	C53—C62—C63—C64	−81.3 (4)
C22—C23—C24—C25	−0.3 (7)	N3—C63—C64—C69	75.9 (4)
C26—C23—C24—C25	−179.6 (4)	C62—C63—C64—C69	−48.2 (4)
C23—C24—C25—C20	0.4 (6)	N3—C63—C64—C65	−101.5 (4)
C21—C20—C25—C24	0.1 (6)	C62—C63—C64—C65	134.4 (4)
C18—C20—C25—C24	178.5 (4)	C69—C64—C65—C66	0.7 (5)
C32—C27—C28—C29	2.1 (5)	C63—C64—C65—C66	178.1 (3)
C36—C27—C28—C29	−178.3 (3)	C64—C65—C66—C67	−2.2 (6)
C27—C28—C29—C30	−0.2 (6)	C65—C66—C67—C68	2.3 (7)
C28—C29—C30—C31	−1.1 (6)	C66—C67—C68—C69	−1.0 (8)
C29—C30—C31—C32	0.4 (6)	C65—C64—C69—C68	0.6 (6)
C28—C27—C32—C33	179.9 (3)	C63—C64—C69—C68	−176.8 (4)
C36—C27—C32—C33	0.4 (5)	C67—C68—C69—C64	−0.5 (7)
C28—C27—C32—C31	−2.8 (5)	N3—C70—C72—C73	−39.3 (5)
C36—C27—C32—C31	177.7 (3)	C71—C70—C72—C73	83.6 (5)
C30—C31—C32—C33	178.8 (4)	N3—C70—C72—C77	144.6 (3)
C30—C31—C32—C27	1.6 (6)	C71—C70—C72—C77	−92.5 (5)
C27—C32—C33—C34	−2.1 (5)	C77—C72—C73—C74	−0.6 (6)
C31—C32—C33—C34	−179.3 (4)	C70—C72—C73—C74	−176.9 (4)
C32—C33—C34—C35	1.6 (6)	C72—C73—C74—C75	1.7 (6)
C33—C34—C35—O1	−178.6 (4)	C73—C74—C75—C76	−1.7 (6)
C33—C34—C35—C36	0.6 (6)	C73—C74—C75—C78	179.4 (4)
O1—C35—C36—C27	177.0 (3)	C74—C75—C76—C77	0.6 (6)
C34—C35—C36—C27	−2.3 (5)	C78—C75—C76—C77	179.5 (4)
O1—C35—C36—C37	−3.0 (5)	C75—C76—C77—C72	0.5 (6)
C34—C35—C36—C37	177.8 (3)	C73—C72—C77—C76	−0.5 (5)
C28—C27—C36—C35	−177.8 (3)	C70—C72—C77—C76	175.8 (3)
C32—C27—C36—C35	1.8 (5)	C20—C18—N2—C7	−59.9 (4)
C28—C27—C36—C37	2.2 (5)	C19—C18—N2—C7	174.8 (3)
C32—C27—C36—C37	−178.3 (3)	C8—C7—N2—C18	−74.3 (4)
C35—C36—C37—N1	−26.7 (4)	C1—C7—N2—C18	160.3 (3)
C27—C36—C37—N1	153.4 (3)	C72—C70—N3—C63	−55.4 (4)
C35—C36—C37—C38	97.3 (4)	C71—C70—N3—C63	179.3 (4)
C27—C36—C37—C38	−82.6 (4)	C62—C63—N3—C70	−84.7 (4)
N1—C37—C38—C39	−104.1 (4)	C64—C63—N3—C70	150.5 (3)
C36—C37—C38—C39	131.2 (4)	C38—C37—N1—C44	156.8 (3)
N1—C37—C38—C43	72.7 (4)	C36—C37—N1—C44	−76.8 (3)
C36—C37—C38—C43	−51.9 (4)	C46—C44—N1—C37	−68.9 (4)
C43—C38—C39—C40	1.3 (6)	C45—C44—N1—C37	166.5 (3)

### Hydrogen-bond geometry (Å, °)

**Table:**

*D*—H···*A*	*D*—H	H···*A*	*D*···*A*	*D*—H···*A*
O1—H1···N1	0.82	1.86	2.571 (4)	144.
O2—H8···N2	0.82	1.92	2.629 (5)	144.
O3—H2···N3	0.82	1.87	2.597 (5)	147.
C6—H53A···O2	0.93	2.58	3.327 (6)	138.
